# Intra-articular Versus Intravenous Tranexamic Acid in Primary Total Knee Replacement

**DOI:** 10.7759/cureus.21052

**Published:** 2022-01-09

**Authors:** Ahmad Furqan, Sohail Hafeez, Fahim Khan, Sajjad H Orakzai, Aamer N Nur, Muhammad A Khan

**Affiliations:** 1 Orthopaedics, Shifa International Hospital, Islamabad, PAK; 2 Orthopedic Surgery, Shifa International Hospital, Islamabad, PAK; 3 Orthopaedics and Traumatology, Shifa International Hospital, Islamabad, PAK; 4 Orthopedics and Traumatology, Shifa International Hospital, Islamabad, PAK; 5 Orthopaedics and Trauma, Shifa International Hospital, Islamabad, PAK

**Keywords:** tranexamic acid, total knee replacement, intra-articular, intra-venous, blood loss, postoperative blood loss

## Abstract

Background

Total knee replacement (TKR) is an artificial joint surgical procedure that replaces the damaged articular surfaces of the knee joint. Despite several studies on the efficacy of intra-articular and intravenous Tranexamic acid (TX) use in reducing blood loss following TKR, the route of TXA administration is still an ongoing topic of debate. Our study aimed to compare total knee replacement efficacy (hemoglobin level, hematocrit level, hospital stay, and complications) of intra-articular and intravenous tranexamic acid administration.

Material and Methods

A Prospective study was conducted at the Department of Orthopedics, Shifa International Hospital, Islamabad. The study duration was six months (August 2020 to February 2021). A sample size of 60 patients was calculated using the WHO calculator. Patients were selected through non-probability consecutive sampling. Patients were randomly divided into two groups; Group A was given intraarticular TXA, while group B was given intra-venous TXA following total knee replacement. Patients were followed for 48 hours. Data were analyzed using SPSS version 24. An Independent T-test was applied, and a P value≤0.05 was considered significant.

Results

A total of 60 patients were included in the study. There were 20 (33.3%) male and female 40 (66.7%). The mean age of patients was 64.4±10.8SD. Post-operative hemoglobin level in group A was 11.09±0.39SD, and in group B was 9.93±1.73SD (p=0.03). Postoperatively, the mean HCT level in group A was 30.53±4.26SD and group B 26.88±5.48SD (p=0.01).

Conclusion

Intra-articular administration of TXA is more effective than intravenous administration in controlling postoperative blood loss following total knee replacement.

## Introduction

Total knee replacement (TKR) is an artificial joint surgical procedure that replaces the damaged knee joint [1]. The primary indications include severe pain causing functional limitation [2]. In the United States, hospitalization due to TKR is increased from 145,242 to 248,267 over six years (2000-2006) [3]. The overall incidence of TKR in the United States is 5.5 to 8.7/1,000 population [4].

Knee replacement prostheses are divided into three major types (Non constrained prosthesis, semi-constrained prosthesis, and constrained prosthesis). Non constrained prosthesis is associated with providing stability to the prosthesis with patients' muscles and ligaments. Semi-constrained is associated with providing stability to the knee without relying on the muscles and ligaments of the patient. At the same time, a constrained prosthesis is used in patients in whom muscles and ligaments cannot provide any stability [5]. TKR can also result in significant blood loss among patients (10-38%) that requires allogeneic blood transfusion (peri-operatively). Blood transfusion might be associated with complications like viral infection, fluid overload, and transfusion-related reactions. The procedure also increases hospital stay and the cost of treatment [6].

Tranexamic acid (TXA) is a synthetic anti-fibrinolytic agent associated with inhibiting the activation of plasminogen to plasmin. TXA at high concentration can also directly inhibit plasmin activity. The process decreases proteolytic action on fibrinogen and fibrin monomers, ultimately preventing clot stabilization. The use of TXA intravenously in TKR is a common practice. Literature reported the use of TKR in the following forms: 1. A pre-operative dose before tourniquet inflation; 2. An intra-operative dose before deflation of the tourniquet; 3. Post-operative dose 3 hours after surgery; 4. Various permutations combining these three doses

Intravenous use of TXA is associated with a wide distribution of the drug in intra and extracellular compartments [7]. Intra-articular use of TXA has gained popularity recently, and it is commonly used as a topical wash or injected into the knee joint through a drain after wound closure. Literature reported that intra-articular use of TXA is associated with ease of administration, minimal systemic absorption, and the ability to achieve maximum concentration at the bleeding site [8].

We found limited literature on the comparison of intra articular and intravenous use of TXA in TKR in Pakistan. The literature available on this topic is not enough to reach any conclusion in the settings of Pakistan. This study was done to cover knowledge gaps regarding the efficacy of tranexamic acid administration routes. So, the present study aims to compare efficacy (Hemoglobin level, hematocrit level, and complications) of intra-articular and intravenous tranexamic acid administration in total knee replacement.

## Materials and methods

**Figure 1:**
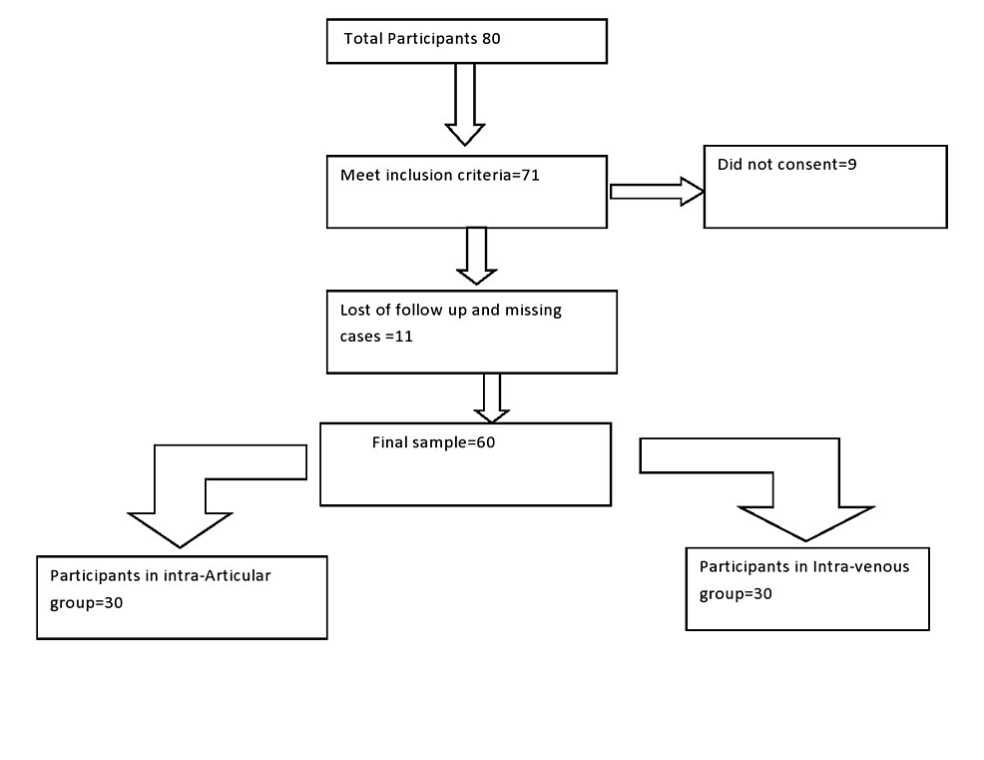
Flow chart of sample selection

A prospective study was conducted at the Department of Orthopedics, Shifa International Hospital, Islamabad. A sample size of 56 was calculated (rounded off to 60), 30 patients in each group using a WHO calculator with µ1=254, µ2=210, SD=60, 95% confidence level, 5% significance level, and 80% power of study [9]. Patients were selected through non-probability consecutive sampling, as shown in figure 1. Ethical approval was taken from the respective department. After explaining the research purpose, risk, and benefits of this research, all participants who were willing to participate signed written consent forms. The department of orthopedics ensured the safety of participants by not using any new drug in the study. The drugs included in the study were already being used in our department. An inclusion criterion was 1) ≥35 years of age; 2) American Society of Anesthesiologists (ASA) score ≤ 3; and 3) planned TKA due to advanced degenerative arthritis of the knee. Exclusion criteria were 1) cardiovascular problems (e.g., myocardial infarction, atrial fibrillation, and angina); 2) cerebrovascular conditions (e.g., previous stroke or previous vascular surgery); 3) thromboembolic disorders; and 4) renal insufficiency. Patients were randomly divided into two groups; Group A received intra-articular TXA while group B received Intra-venous TXA. Both interventional groups underwent a conventional bilateral total knee replacement. In group A, patients were given 1.5gram of TXA in 100cc of normal saline directly in the knee joint cavity during suturing (immediately after deflation of tourniquet). In group B, 1.5 grams of TXA in 100cc of normal saline was given intravenously immediately after surgical site closing (after deflation of tourniquet). We gave a single injection in both groups. Two experienced surgeons performed surgeries of both groups. Patients were followed for 48 hours postoperatively. Primary outcomes of the study were differences in pre and post-operative hemoglobin level and hematocrit level, while secondary outcomes were hospital stay and complications.

Data analysis

Data analysis was done with SPSS version 24. Descriptive statistics include the presentation of mean±standard deviation, frequency, and percentages. Inferential statistics will include an independent t-test, and P-value ≤0.05 was considered as a statistically significant finding.

## Results

**Figure 2:**
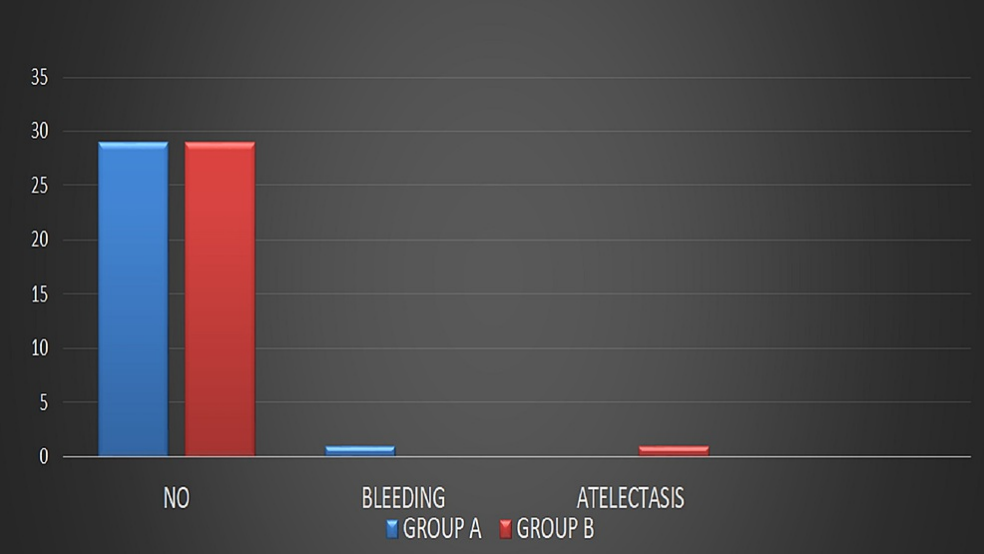
Comparison of Complications in both groups

A total of 60 patients were included in the study. There were 20 (33.3%) male and female 40(66.7%) patients. The mean age of patients was 64.4±10.8SD. There were 10(16.7%) patients in the age group 40-50 years and 50(83.3%) in the age group >50 years. There were two interventional groups; group A 30 (5-%), and group B, 30(50%) patients. Pre-operative hemoglobin level in group A was 12.62±1.14SD and in Group B 12.53±1.41SD (p= 0.780). The post-operative hemoglobin level in group A was 11.09±0.39SD, and group B was 9.93±1.73SD (p=0.03), as shown in table 1. The pre-operative mean HCT level in group A was 38.63±2.77SD and in group B 37.87±4.19SD (p=0.412). The mean HCT level postoperatively in group A was 30.53±4.26SD and group B 26.88±5.48SD (p=0.01), as shown in table 2. Mean hospital stay in group A was 3.4±1.3SD, and group B was 5.5±3.2SD (p=0.01), as shown in table 3. No significant difference in complications of both groups was reported (p=0.652), as shown in figure 2.

**Table 1:** Comparison of pre and post-operative hemoglobin levels in both groups TXA- Tranexamic acid.

Interventional groups	N=60	Pre-operative hemoglobin level		P value
		Mean	Standard deviation	
Group A (IA-TXA)	30	12.623	1.145	0.780
Group B (IV-TXA)	30	12.53	1.41	
		Post-operative hemoglobin level		
		Mean	Standard deviation	
Group A (IA-TXA)	30	11.09	0.39	0.03
Group B (IV-TXA)	30	9.93	1.73	

**Table 2:** Comparison of pre and post-operative hematocrit level in both interventional groups TXA- Tranexamic acid.

Interventional groups	N=60	Pre-operative hematocrit level		P value
		Mean	Standard deviation	
Group A (IA-TXA)	30	38.63	2.77	0.412
Group B (IV-TXA)	30	37.87	4.19	
		Post-operative hematocrit level		
		Mean	Standard deviation	
Group A (IA-TXA)	30	30.53	4.26	0.01
Group B (IV-TXA)	30	26.88	5.48	

**Table 3:** Comparison of hospital stay duration in both interventional groups TXA- Tranexamic acid.

Interventional groups	N=60	Hospital stay (Days)	P value
		Mean ±SD	
Group A (IA-TXA)	30	3.4±1.3	0.01
Group B (IV-TXA)	30	5.5± 3.2	

## Discussion

Total knee replacement is a cost-effective treatment modality for advanced degenerative arthritis of the knee [10]. The major complication of TKR is blood loss pre and postoperatively, which requires substantial blood transfusions [11]. Despite several studies on the efficacy of intra-articular and intravenous TXA use in reducing blood loss following TKR, the route of TXA administration is still an ongoing topic of debate.

In our study, the intra-articular route showed less blood loss in hemoglobin and hematocrit levels and less hospital stay (p<0.05) than intravenous administration of TXA. Huang et al. reported that intra articular and intravenous routes effectively reduce blood loss after TKR [12]. However, Hamlin et al. reported that the intra-articular route of administration is more likely to be associated with less blood loss and reduced need for post-operative blood transfusions than the intravenous route [13]. While Seo et al. also reported similar findings in favor of the intra-articular route of administration [14].

Some studies also supported the postulation that TXA showed a more effective therapeutic effect after proteolysis of plasmin dissolved prematurely fibrin clot. TXA showed a more effective response at the active bleeding site of the wound than within blood vessels [15]. Moreover, evidence of conflicting findings also exists depending upon variation in surgical technique (using conventional intra and extramedullary jigs or computer-assisted surgery), variation in dosage (some studies use a single dose of TXA while other use three doses), and variation in blood transfusion indications across different hospitals [16].

Our study did not find any significant difference in complications in both groups. However, Wang et al. promptly reported that the intravenous route is associated with more complications, including surgical site infection and bleeding [17]. Moskal et al. reported that the intra-articular route of administration is associated with minor complications, including bleeding and atelectasis [18]. Patel et al. reported no significant difference in a post-operative complication of TXA administration through intra-articular and intravenous routes [19]. Yang et al. reported that both groups are associated with some minor blood loss complications [20].

Strength: To the best of our knowledge, it is a unique study in our local setup. It is challenging to conduct these studies as very few centers perform TKR in such numbers.

Limitation: Small sample size and conduction of study at a single center limits generalization of results.

## Conclusions

Intraarticular administration of TXA is more effective than intravenous administration in controlling postoperative blood loss following total knee replacement. Our study did not find any significant difference in complication after intra-articular and intravenous administration of TXA. More detailed, multicenter clinical trials are required to understand the efficacy and cost of treatment in-depth.
